# Development of camel and cow's milk, low‐fat frozen yoghurt incorporated with Qodume Shahri (*Lepidium perfoliatum*) and cress seeds (*Lepidium sativum*) gum: Flow behavior, textural, and sensory attributes' assessment

**DOI:** 10.1002/fsn3.2139

**Published:** 2021-01-21

**Authors:** Mojtaba Azari‐Anpar, Morteza Khomeiri, Amir Daraei Garmakhany, Sorour Lotfi‐Shirazi

**Affiliations:** ^1^ Department of Food Science and Technology Faculty of Agriculture Ferdowsi University of Mashhad Mashhad Iran; ^2^ Université Claude Bernard Lyon 1 ISARA Lyon Univ Lyon Bourg en Bresse France; ^3^ Department of Food Science & Technology Gorgan University of Agricultural Sciences & Natural Resources Gorgan Iran; ^4^ Department of Food Science and Technology Tuyserkan Faculty of Engineering & Natural Resources Bu‐Ali Sina University Hamedan Iran

**Keywords:** camel milk, cress seed gum (*Lepidium sativum*), frozen yoghurt, Qodume Shahri seed gum (*Lepidium perfoliatum*), rheological properties

## Abstract

In this study, the effect of different concentrations (0.2%, 0.1%, and 0%) of some plant seed gums (Qodume Shahri (*Lepidium perfoliatum*) and cress (*Lepidium sativum*)) as the stabilizer on the flow behavior, textural, and sensory properties of frozen yoghurt produced from camel and cow's milk was investigated. The results showed that plant seed gums significantly affected the viscosity, overrun and melting rate, textural, and sensory properties of frozen yoghurt samples prepared from camel and cow's milk. Also, no significant differences were observed in pH and acidity of the samples. The highest overrun value was observed in the control sample. Frozen yoghurt containing 0.2% Qodume Shahri seed gum (QSSG) had the highest viscosity and the longest first dripping time. This is an indication that frozen yoghurt mixes are non‐Newtonian at all added concentrations. Finally, Herschel–Bulkley model well described the rheological behavior of frozen yoghurt mixtures due to the higher correlation coefficient. In general, cow's frozen yoghurts containing 0.2% cress seed gum (CSG) and 0.1% QSSG were more acceptable among panelists than camel frozen yoghurt sample.

## Graphical abstract

The effect of different concentrations (0.2%, 0.1%, and 0%) of some plant seed gums (Qodume shahri (*Lepidium perfoliatum*) and cress (*Lepidium sativum*)) as the stabilizer on the flow behavior, textural, and sensory properties of frozen yoghurt produced from camel and cow's milk was investigated in this study.

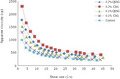

## 1 INTRODUCTION

Camel milk is one of the very important source of nutrient for human in numerous arid and semiarid environment (Agrawal et al., 2011), and it has been used in the treatment of many diseases including the tuberculosis and other lung ailments in Russia, dropsy, jaundice, and anemia in India (Hashim et al., 2009) and finally glycemic control and reduced insulin dose in patients with type‐I diabetes (Agrawal et al., 2011). Camel milk is a complex mixture of total solid (9.8%–14.4% w/w), fat (3.2%–5.5% w/w), protein (2.7%–4.5% w/w), lactose (3.4%–5.5% w/w), and ash (0.6%–0.9% w/w) (Tamime & Robinson, 2013). Since the physicochemical properties of camel milk differ from those of cow's milk, thus, the products manufactured from camel milk can be different from cow's milk. Usually, camel milk is consumed fresh or fermented (Singh et al., 2017). Frozen dairy desserts can be made from milk of different animals such as cow's and camel milk (Kavas & Kavas, 2016), sheep's milk (Elisangela et al., 2016), and goat's milk (Ranadheera et al., 2013).

Frozen yoghurt is a refreshing and nutritious dessert, with or without the flavor that combines the texture of ice cream and yoghurt (Goff, 2018). Like most frozen dairy desserts, frozen yoghurt contains milk fat, milk solids‐not‐fat, sweetener, stabilizer, emulsifier, and water. It is low in fat, typically 2%–4% (Goff & Hartel, 2013).

Furthermore, there are many factors that influence the body and texture of the ice cream and similar products. Stabilizers are one of the main ingredients that have important functions on the final product quality. For example, they are used in the ice cream mixture to increase mixture viscosity; prevent the separation of clear serum during meltdown; and restrict the growth of ice and lactose crystals during storage periods, especially during periods of temperature fluctuation. They also produce a stable foam with easy cutoff and stiffness in the barrel freezer for packaging; provide uniformity to the product and increase resistance to melting; and finally improve the feeling of smoothness texture during consumption in the final product (Clarke, 2012; Goff & Hartel, 2013).

Recently, the trend in the enrichment of the dairy products with herbal extract particularly food hydrocolloids from natural gums obtained from various sources with different physical and functional properties in the food industry has been increased significantly (Behrouzian et al., 2014; Mirhosseini & Tab atabaee Amid, 2012). Fortified food with the herbal additives can be unique and provide more health benefits (Panesar & Shinde, 2012). *Lepidium perfoliatum* and *Lepidium sativum* both types of herb belong to the Cruciferae family where is originated from Iran and locally called Qodume Shahri and cress, respectively. The parts of the plant which are used are the roots, leaves, and seeds (Amin, 2005). The *L. perfoliatum* and *L. sativum* seeds contain a large amount of mucilaginous substances which diffuses out when soaked in water under optimal conditions (Seyedi et al., 2014). Therefore, the aim of the present study was to investigate the possibility of producing low‐fat frozen yoghurt from camel and cow's milk by applying these gums and comparing the rheological, textural, and sensory characteristics of produced low‐fat frozen yoghurt samples.

## 2 MATERIALS AND METHODS

### 2.1 Extraction procedure of QSSG and CSG gums

The Qodume Shahri and cress seeds were purchased from the Attari, herbal medicine shop, in Gorgan, Iran. The seeds were cleaned by hand to remove all foreign material. The Qodume Shahri seed gum (QSSG) and cress seed gum (CSG) were extracted under optimal conditions (pH = 8; 48°C and water to seed ratio of 30 (ml):1 (g); pH = 10; 35°C, at a water to seed ratio of 30 (ml):1 (g), respectively). The pH was adjusted constantly by the addition of 0.1 mol/L NaOH and/or HCl. The seed‐water slurry was stirred with an electric stirrer throughout the entire extraction period (1.5–3.5 hr); then, the seeds were discarded, and remained gum slurry was dried to overnight by oven at 50 ± 1°C. The obtained powder was milled and sieved by using a mesh 18 sifter to get the same particles then packed and stored in cool and dry conditions (Azari‐Anpar, et al., 2017; Razavi et al., 2011).

### 2.2 Frozen yoghurt preparation

Fresh whole camel (total solid (13.18% w/w), fat (4.1% w/w), protein (3% w/w), lactose (4.4% w/w), and ash (0.88% w/w) and cow's milk (total solid (12.58% w/w), fat (3.8% w/w), protein (3.2% w/w), lactose (4.87% w/w), and ash (0.88% w/w) were obtained from the Gorgan province of Iran. Both types of milk were standardized using skim milk powder to 11% SNF, according to the Iranian standard for yoghurt (ISIRI, NO: 695, 4th revision, 2005). Milk was heated at 85°C for 30 min and cooled to approximately 42°C, then inoculated with a commercial yoghurt culture (YC‐X11; CHR‐Hansen, Horsholm, Denmark), kept at this temperature for 4 hr, and finally transferred to refrigerator temperature.

**FIGURE 1:**
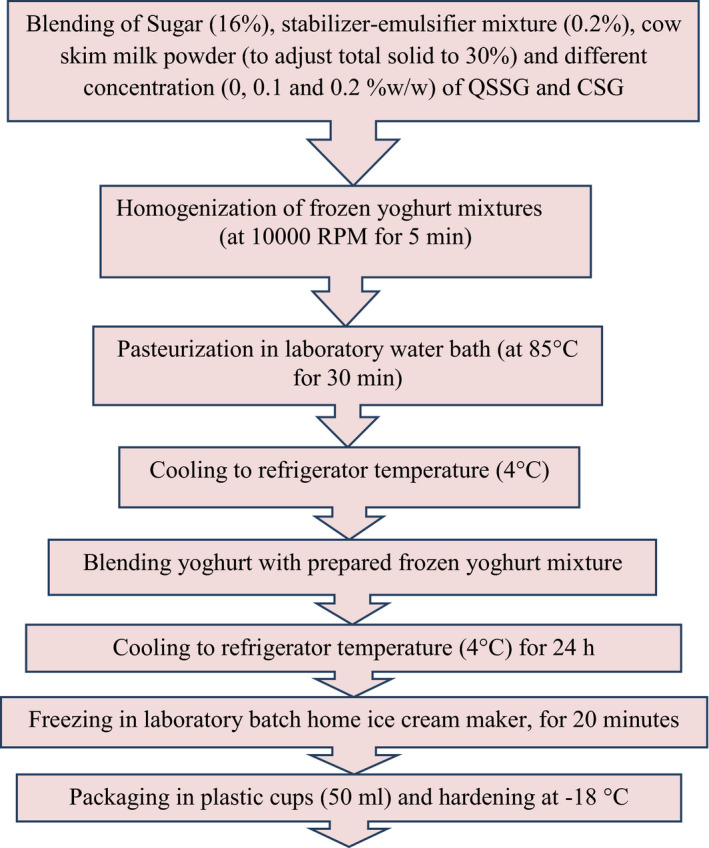
The flow diagram of low‐fat frozen yoghurt production

The flow diagram of frozen yoghurt manufacturing stages is presented in Figure 1 and described as below (Rezaei et al., 2011):

– Blending of Sugar (16%), stabilizer‐emulsifier mixture (0.2%), cow skim milk powder (to adjust total solid to 30%), and different concentration (0, 0.1, and 0.2% w/w) of QSSG and CSG (depending on the type of treatment for both types of milk).
– Homogenization of frozen yoghurt mixtures (Heidolph homogenizer, made in Germany) (at 1344 *g* for 5 min).
– Pasteurization in laboratory water bath (at 85°C for 30 min).
– Cooling to refrigerator temperature (4°C).
– Blending yoghurt with prepared frozen yoghurt mixture.
– Cooling to refrigerator temperature (4°C) for 24 hr (to ensure complete hydration of the mixed ingredients).
– Freezing in laboratory batch home ice cream maker (Musso, made in Italy) having 1.5 L capacity, for 20 min.
– Packaging in plastic cups (50 ml) and hardening at −18°C.

### 2.3 The pH and acidity measurement

The pH was measured using a Metrohm‐827 pH meter (Metrohm, Herisau, Switzerland) after calibration using commercial pH 4.00 and 7.00 buffers. The titratable acidity of aged frozen yoghurt mixtures was determined according to National Standard of Iran (ISIRI, NO: 2852, 4th revision, 2006) by titration using 0.1 N standardized NaOH in the presence of phenolphthalein and the results expressed as a percentage of lactic acid in the samples (Reyahi‐Khoram et al., 2018).

### 2.4 Viscosity and flow behavior characteristic of frozen yoghurt mixture

After aging process (kept at 5°C overnight), the apparent viscosity of the prepared frozen yoghurt mixtures made by different concentration of two gums was measured using a Brookfield Viscometer (RV‐DV II Brookfield, Middleboro, MA, USA). Samples were tested in triplicate using spindle No. 5 at speed 100 rpm at 5°C. To determine the rheological behavior of samples, shear rate and shear stress values (at rotation speeds of 10–200 rpm) were calculated using obtained data from the viscometer according to Mitchka's equations (Azari‐Anpar, Khomeiri, et al., 2017; Azari‐Anpar, et al., 2017; Rezaei et al., 2011). The Power Law model (Equation 1), Herschel–Bulkley model (Equation 2), and Casson model (Equation 3) were used to calculate the consistency index (*k*), the flow behavior index (*n*), yield stress (*τ*_0_), and correlation coefficient (*R* ^2^) of samples. Finally, the experimental data were fitted according to these three models.

Power law model:

(1) $$\tau =k{\left(\gamma \right)}^{n}$$

Herschel–Bulkley model:

(2) $$\tau =\tau_{0}+k{\left(\gamma \right)}^{n}$$

Casson model:

(3) $$\tau^{0.5}=\tau_{0}^{0.5}+k{\left(\gamma \right)}^{0.5}$$

where *τ* is shear stress (Pa), *γ* is the shear rate (s^−1^), *k* is the consistency coefficient (Pa s*^n^*), *n* is the flow behavior index (dimension less), and *τ*_0_ is yield stress (Pa). For shear‐thinning fluids, *n* < 1 and for shear‐thickening fluids, *n* > 1.

### 2.5 Overrun

The overrun of frozen yoghurt samples was calculated using the following equation (Akın et al., 2007; Azari‐Anpar, Khomeiri, et al., 2017).

(4) $$\text{Overrun}\;\left(\%\right)\;=\frac{\left(W_{1}‐W_{2}\right)}{W_{2}}\times 100$$

where *W*_1_ = the weight of unit mixture; *W*_2_ = the weight of same volume of frozen yoghurt.

### 2.6 Melting characteristics

Melting behavior expressed as the first (1st) dripping time and melting rate was evaluated on frozen yoghurt samples stored after 1 day at −18°C. Blocks of frozen yoghurt with weight of 30 ± 1 g were placed on a sieve with a mesh diameter of 2 mm and incubated at 25 ± 1°C. After 60 min, depending on the weight of the melted liquid, melting rate was measured as a percentage of initial weight. The time of the first drop was recorded during melting process (Akalın & Erişir, 2008).

### 2.7 Textural analysis

Instrumental texture profile analysis (TPA) was performed on the frozen yoghurt samples using a Texture Analyzer TA‐XT‐plus (Stable Micro Systems, Surrey, UK), equipped with a 2 mm diameter stainless steel cylindrical probe and set up to record the desired forces for penetrating distance = 15 mm, force = 5 g, probe speed during penetration = 3.3 mm/s, probe speed pre‐ and postpenetration = 3 mm/s. Factors evaluated in this test were hardness (maximum force during penetration), adhesiveness (negative area of peak force during withdrawal), gumminess (hardness × cohesiveness (that describes how well a food retains its form between the 1st and 2nd chew)), and chewiness (springiness (that is a textural parameter, which is related to elasticity of the sample) × gumminess (that describes being soft and sticky)). Before testing, samples were stored at −10°C for 24 hr (Akalın et al., 2008).

### 2.8 Sensory evaluation of frozen yoghurt

Evaluation of sensory properties of the produced frozen yoghurt was done, after maintenance at −18°C for 1 day. All samples were tempered for 5 min at 20 ± 2°C prior to sensory testing. In order to evaluate sensory attributes, 8 trained panelists were tested the samples. After initial training of the panelists to acquaint the desired properties, the samples were evaluated in term of texture, flavor, color, and total acceptability (Azari‐Anpar, et al., 2017; Mehrinejad Choobari et al., 2020). In this test, the 5‐point hedonic scale (excellent sample = 5, good = 4, average = 3, bad = 2, and too bad = 1) was used.

### 2.9 Statistical analysis

In order to evaluate obtained physical and chemical properties, results were analyzed based on a completely randomized design by using SPSS software (version 21). Average data were compared using the Duncan multiple range test at the 5% level, and graphs were plotted using Microsoft Excel (version 2013) and Curve Expert 1.3. All tests and experiments were performed in three replicates.

## 3 RESULTS AND DISCUSSION

### 3.1 pH and acidity

As shown in Table 1, no significant differences (*p* > .05) were observed on the pH and acidity of frozen yoghurt samples containing gums. Results showed that the pH ranged from 5.23 to 5.42 and the acidity ranged from 7.5 to 7.65 as a percentage of lactic acid. These observations are in agreement with the study of Rezaei et al. (2011) and Bahramparvar et al. (2009) which showed that the addition of stabilizers had no effect on pH and acidity of samples. As presented in Table 1, there are significant difference between produced yoghurt from camel and cow milk in terms of the pH and acidity which are in agreement with the results of Al‐Saleh et al. (2011). That may be due to the differences in buffering capacities between the mixes made from cow's and camel milk. Generally, the total amount of protein in cow's milk is more than camel milk; therefore as a result, the buffering capacity of cow's milk can be higher than the camel milk.

**TABLE 1:** Effect of QSSG and CSG on the physicochemical properties of cow's and camel frozen yoghurt (*n* = 3)

Frozen yoghurt	Gum concentration (%)	pH	Acidity	Overrun (%)	First dripping time (s)	Melting rate (%)	Viscosity (cp)
Cow's milk	Control	5.24 ± 0.01^a^	7.65 ± 0.01^a^	39.35 ± 0.9^d^	1,165 ± 22.6^h^	75.9 ± 0.66^c^	1,005.3 ± 5.03^e^
	0.1% QSSG	5.23 ± 0.02^a^	7.64 ± 0.01^a^	31.2 ± 1.7^g^	1,863 ± 23.3^c^	46.47 ± 1.50^f^	1,713.7 ± 22.2^c^
	0.2% QSSG	5.24 ± 0.01^a^	7.64 ± 0.01^a^	20.13 ± 0.6^i^	2,174.3 ± 3.7^a^	17.21 ± 3.00^h^	2,138.3 ± 63.4^a^
	0.1% CSG	5.24 ± 0.01^a^	7.63 ± 0.01^a^	37.3 ± 1.8^e^	1,676 ± 5.29^e^	65.49 ± 4.41^e^	1,636 ± 10.6^d^
	0.2% CSG	5.24 ± 0.01^a^	7.65 ± 0.02^a^	26.42 ± 1.9^h^	1,969.3 ± 10^b^	40.27 ± 0.42^g^	1,889.7 ± 5.5^b^
Camel milk	Control	5.40 ± 0.00^b^	7.51 ± 0.01^b^	54.47 ± 0.6^a^	928.6 ± 3.2^i^	96.01 ± 0.54^a^	299 ± 7.6^j^
	0.1% QSSG	5.42 ± 0.01^b^	7.51 ± 0.01^b^	44.54 ± 0.6^c^	1,336.3 ± 25.1^g^	79 ± 1.07^c^	437 ± 7.5^h^
	0.2% QSSG	5.41 ± 0.01^b^	7.51 ± 0.21^b^	24.95 ± 0.3^h^	1,771.6 ± 5^d^	47.85 ± 0.87^f^	630 ± 40^f^
	0.1% CSG	5.40 ± 0.01^b^	7.51 ± 0.01^b^	49.51 ± 1.2^b^	1,184 ± 8.7^h^	88.55 ± 0.81^b^	355 ± 5.0^i^
	0.2% CSG	5.42 ± 0.01^b^	7.50 ± 0.01^b^	33.81 ± 0.9^f^	1,479 ± 14.9^f^	69.44 ± 2.24^d^	516 ± 4.0^g^

Note

In each column, means with same superscripts had no significant difference with each other (*p* > .05).

### 3.2 Overrun

The amount of overrun of frozen yoghurt samples was different from 20.13% to 54.47% (Table 1). The type of milk, type and concentration of gums had a significant effect on the amount of overrun (*p* < .05). Results showed that in all samples, the amount of overrun was reduced with increasing the stabilizers' concentration. Cow's frozen yoghurt containing 0.2% QSSG and control sample made from camel milk (without gum) had lowest and highest overrun values than the other samples, respectively. Also, QSSG is more effective in reducing the overrun than CSG in the same concentration (*p* < .05) for both frozen yoghurt produced from cow and camel milk. One of the most important properties of the stabilizers is increasing ice cream volume through increasing the viscosity by keeping the air bubbles (Bahramparvar & Mazaheri Tehrani, 2011). But this trend was not observed in the present study. Akalın et al. (2008) and Bahramparvar et al. (2009) mentioned that reducing the overrun can be due to the lack of efficiency of batch type freezers. On the other hand, it seems that along with increasing concentrations of gums and during the whipping process and freezing the air cells has not been able to fit into the frozen yoghurt body and they prevented from the appropriate distribution.

In addition, the overrun values for camel milk ranged from 24.95% to 54.47% and for cow's milk were between 20.13% and 39.35%. Abu‐Lehia et al. (1989) reported that the physicochemical properties of ice cream mixtures are dependent on the milk type and milk composition. Also, the viscosity of camel frozen yoghurt mixtures is lower than cow's milk where this issue allows to air easily incorporated with camel frozen yoghurt mixtures.

### 3.3 Melting properties

Besides overrun, the melting rate also is an important quality index for frozen dairy dessert. The amount of melting rate and first dripping time are presented in Table 1. As can be seen, applied treatments have different melting properties (*p* < .05). Results showed an inverse relationship between the results of melting properties and overrun, as overrun decrease, melting resistance of samples enhances. Cow's frozen yoghurt containing 0.2% QSSG has the highest (17.21%), and camel control samples have the lowest (96.01%) resistance to melting. One of the most important functions of a stabilizer in ice cream products is improving the melting properties. Stabilizers due to their water‐holding and microviscosity enhancement ability increase the melting resistance of frozen yoghurt (Bahramparvar & Mazaheri Tehrani, 2011). In this case, other researchers reported that the addition of stabilizers not only increased ice cream mix viscosity, but also resulted in a lower melting rate and consequently improved the melting resistance (Milani & Koocheki, 2011; Moeenfard & Tehrani, 2008).

The melting rate of the frozen yoghurt made from camel milk was significantly higher than the produced frozen yoghurt from cow's milk with the same values (*p* < .05). These results also may be due to the fact that yoghurt made from camel milk had a fragile structure compared to the yoghurt made from cow's milk, which forms a weak network around of the air cells and lead to decrease meltdown resistance in these samples.

First dripping time was influenced by both the gums and type of milk (*p* < .05). The first dripping time values for cow's frozen yoghurt samples, ranged from 1,165 to 2,174.3 s and for frozen yoghurt samples produced from camel milk were between 928 to 1,771 s, depending on the type of gum and type of applied milk (Table 1). The first dripping time appeared after 928 s for camel frozen yoghurt (without gums), while it was observed after 2,174 s for cow's frozen yoghurt (containing 0.2% QSSG). The increase of gum concentration leads to the increase of first dripping time of frozen yoghurt. Stabilizers can form a sticky network in yoghurt ice cream that results in more resistance to melting (El‐Nagar et al., 2002). The same reasons that mentioned for melting rate is also true for the first dripping time of the frozen yoghurt. According to Milani and Koocheki (2011) and Rezaei et al. (2011), hydrocolloid stabilizers lead to an increase in the first dripping time.

### 3.4 Rheological properties

#### 3.4.1 Viscosity

The apparent viscosity of frozen yoghurts mixtures made from both types of milk (cow and camel) is shown in Table 1. There were significant differences between all mixtures in term of viscosity (*p* < .05). As can be seen, the apparent viscosity of frozen yoghurt mixtures was enhanced by increasing the gum concentration. Addition of QSSG and CSG lead to different apparent viscosity and samples containing QSSG had the higher apparent viscosity than samples containing CSG.

Several studies were conducted on the both types of gums previously. They found that the apparent viscosity of produced samples from QSSG was higher than CSG. These results may be related to the chemical structure and molecular weight of these gums (Karazhiyan et al., 2011; Koocheki et al., 2013; Razavi et al., 2011). Basically, whatever the size and molecular weight of hydrocolloid is high, the friction between the molecules and so the viscosity will be the greater (Razavi et al., 2011). Similarly, Rezaei et al. (2011) investigated the effects of guar and arabic gum on the physicochemical characteristics of frozen yoghurt. They reported that by increasing the amount of both gums the viscosity of frozen yoghurt mixtures increased. Also other researches, have studied the effects of QSSG and CSG (Azari‐Anpar, Soltani Tehrani, et al., 2017), resistant starch and maltodextrin (Azari‐Anpar, Khomeiri, et al., 2017), *Lallemantia royleana* (Balangu) seed gum (Bahramparvar et al., 2009), *Gundelia tournefortii* L. seed gum (Cakmakci & Dagdemir, 2013), on the physicochemical properties of ice cream. They concluded that, stabilizers depending on the type and their concentration has a high water‐holding capacity, which can lead to increase the mixture viscosity.

On the other hand, the cow's frozen yoghurt mixture had higher apparent viscosity (Table 1) than camel frozen yoghurt mixture at the equal concentration of stabilizers. One of the main reasons for this, is that, camel milk coagulate cannot be formed correctly. Hashim et al. (2009) reported that the presence of antibacterial agents (lysozyme, lactoferrin, lactoperoxidase, immunoglobulin G, and secretary immunoglobulin A) in camel milk shows inhibitory activity against the starter culture growth. Since these antibacterial agents have a high resistance to heat treatment compared to those in cow's milk, lead to make up a fragile coagulum. Therefore, it can be easily stated that the viscosity of cow's frozen yoghurt mixtures had higher than camel milk (Table 1).

#### 3.4.2 Flow behavior

**FIGURE 2:**
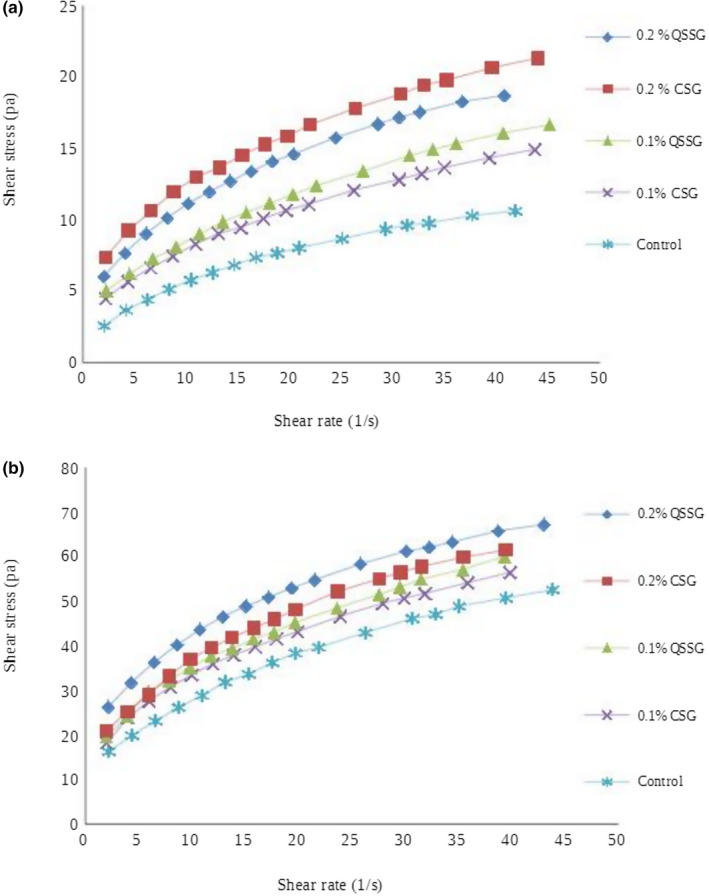
(a) The effect of QSSG and CSG on the flow curve of camel frozen yoghurts. (b) The effect of QSSG and CSG on the flow curve of cow frozen yoghurts

**FIGURE 3:**
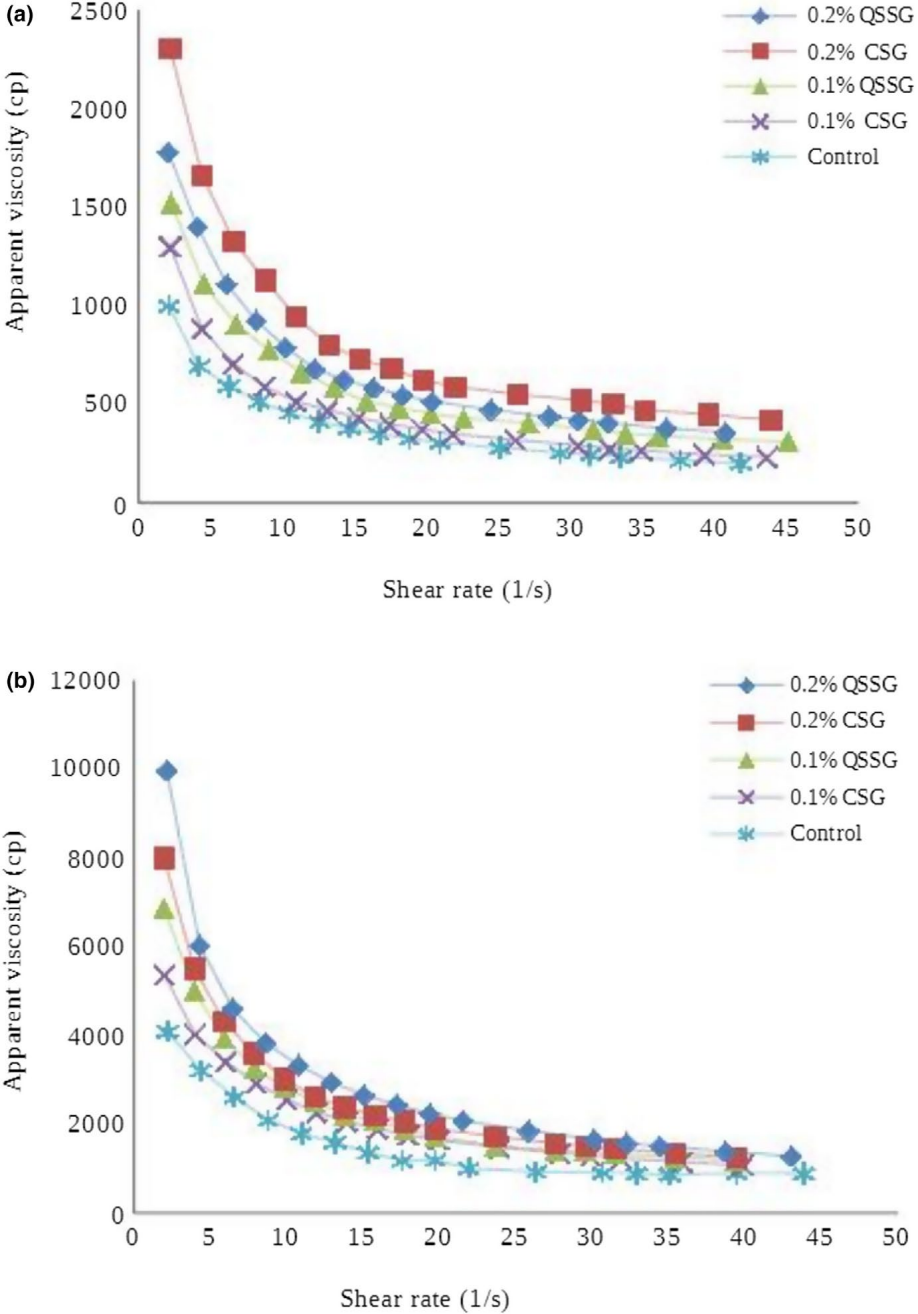
(a) The effect of QSSG and CSG on the apparent viscosity of camel frozen yoghurt. (b) The effect of QSSG and CSG on the apparent viscosity of cow frozen yoghurt

The Figures 2a,b and 3a,b showed the data curve of the shear stress versus shear rate and apparent viscosity versus shear rate in camel and cow's frozen yoghurts containing QSSG and CSG, respectively. The Herschel–Bulkley, Power Law, and Casson models adequately were fitted the data curve for all mixtures. Consistency coefficients (*k*) and flow behavior index (*n*) are also shown in Table 2. It could be seen that, the flow behavior index values for frozen yoghurt mixture samples, exhibited non‐Newtonian behavior. The flow behavior of fluid foods is characterized in terms of the values of *n* and *k* quantities, therefore these parameters (*n* and *k*) are two important factors in rheological properties of fluid foods (Bahramparvar & Mazaheri Tehrani, 2011).

**TABLE 2:** Rheological parameters of the flow curves of the frozen yoghurt containing QSSG and CSG

Frozen yoghurt	Gum concentration (%)	Herschel–Bulkley model				Power Law model			Casson model		
		*τ*_0_	*k*	*n*	*R* ^2^	*k*	*n*	*R* ^2^	*k*	*τ*_0_	*R* ^2^
Cow's milk	Control	3.09	8.53	0.47	0.999	10.65	0.43	0.998	0.43	8.1	0.997
	0.1% QSSG	4.52	11.28	0.43	0.999	14.64	0.38	0.999	0.40	11.6	0.997
	0.2% QSSG	4.04	23.55	0.30	0.999	20.22	0.32	0.999	4.77	16.40	0.995
	0.1% CSG	0.40	13.73	0.38	0.999	14.03	0.38	0.999	0.34	10.9	0.997
	0.2% CSG	0.70	14.70	0.39	0.998	15.23	0.38	0.998	0.43	12.3	0.995
Camel milk	Control	1.80	3.27	0.36	0.999	1.97	0.46	0.998	0.02	0.60	0.997
	0.1% QSSG	1.62	2.12	0.52	0.999	3.18	0.44	0.998	0.03	1.33	0.999
	0.2% QSSG	0.92	4.70	0.39	0.999	5.39	0.36	0.999	0.00	2.37	0.987
	0.1% CSG	0.95	2.37	0.47	0.999	3.02	0.42	0.999	1.53	1.14	0.998
	0.2% CSG	0.73	5.08	0.37	0.999	4.53	0.39	0.999	1.63	2.15	0.964

Abbreviations: *k*, consistency coefficient (Pa s*^n^*); *n*, flow behavior (dimensionless); *R* ^2^ = correlation coefficient; *τ*_0_, yield stress (Pa).

According to the obtained results in this work, *n* values of the frozen yoghurt mixtures were in the range of 0.30–0.52 in applied models. Goff and Hartel (2013) reported that the flow behavior index (*n*) of ice cream mixtures is around 0.7. Also, it should be noted that other investigators have found the flow behavior index values from 0.37 to 0.98 for Power Law model (Bahramparvar et al., 2009). Based on this study, the values of flow behavior index obtained from sample mixtures were closer to the reported values. In general, the obtained results from frozen yoghurt mixtures showed that the value of consistency coefficient (*k*) increased with increasing gum concentration. The increase in the value of consistency coefficient along with gum concentration may be due to an increase in water binding capacity of the gums. As expected, by increasing gum concentration, pseudoplasticity or shear‐thinning behavior of the final product was increased (nonlinear relationship between shear stress and shear rate, with the apparent viscosity decreasing by increasing shear rate). The pseudoplasticity (decreases *n* values) might be associated with the increased alignment of the constituent molecules of the system (Bahramparvar & Mazaheri Tehrani, 2011). Karazhiyan et al. (2009) and Koocheki et al. (2013) reported that strong shear‐thinning behavior of samples containing QSSG and CSG may be due to two origins: firstly enhanced macromolecular entanglement due to the relatively rigid chain conformation of gums and secondly the presence of gel‐like structure which is due to the tendency of molecular association.

Among three studied model, Herschel–Bulkley model well described the rheological behavior of frozen yoghurt mixtures due to its higher correlation coefficient (*R* ^2^).

The flow properties of *Lepidium perfoliatum* gum, extracted from Qodume Shahri seeds and *Lepidium sativum* gum, extracted from cress seeds as a function of the concentrations were investigated by Koocheki et al. (2013) and Karazhiyan et al. (2011) respectively. They showed that, these extracts exhibited strong shear‐thinning behavior and Qodume Shahri extract showed the sharper shear‐thinning behavior compared to cress seed extract. Several studies have investigated the rheological properties of frozen dairy desserts by various stabilizers, which is in agreement with the results of this study (Cakmakci & Dagdemir, 2013; Karazhiyan et al., 2011; Koocheki et al., 2013).

Since, production of fermented products from camel milk is difficult because of the problem of milk coagulation, therefore frozen yoghurts made from camel milk in contrast to cow's milk has lower viscosity (Figure 2). Camel milk has a similar protein content, lower lactose and fat content with saturated fatty acids and greater total cholesterol compared to cow's milk (Hashim et al., 2009). Thus, strong shear‐thinning behavior in cow's frozen yoghurt than camel frozen yoghurt might be due to the interaction between gums and milk components in both milk types that are different from each other.

### 3.5 Textural analysis

#### 3.5.1 Hardness

Hardness is another very important property that directly affects the frozen products final quality (degree of smoothness, intensity of coldness, appearance and overall acceptability). Table 3 presents the effect of QSSG and CSG on the hardness amount of frozen yoghurt made from cow's and camel milk. As can be seen, such as other factors cow's frozen yoghurt containing 0.2% QSSG and control frozen yoghurt made from camel milk (without gum) have the highest (124.6 ± 0.8) and lowest (46.5 ± 1.2) hardness, respectively. Numerous studies have found that, increasing stabilizers concentration increase the hardness of the frozen yoghurt sample. Increasing the gum concentration led to increase apparent viscosity of the serum phase of the ice cream mixture, mostly due to the high water‐holding capacity, which led to increase of the hardness of the final frozen yoghurt product (Azari‐Anpar, Soltani Tehrani, et al., 2017). Another factor has been found to influence hardness of the ice cream is the overrun (Goff & Hartel, 2013). The results of this study showed that the overrun of sample decreased by increase gum concentration. Goff and Hartel (2013) have found that increase overrun lead to decreases the hardness of the final ice cream. Similarly, Martinou‐Voulasiki and Zerfiridis (1990) also reported that the addition of natural stabilizers increased the hardness of frozen yoghurt ice cream. As a result, the presence of a QSSG and CSG improved the hardness of frozen yoghurt with increasing apparent viscosity (Rezaei et al., 2015).

**TABLE 3:** Effect of QSSG and CSG on the textural properties of cow's and camel frozen yoghurt (*n* = 3)

Frozen yoghurt	Gum concentration (%)	Hardness (g)	Adhesiveness (g.sec)	Gumminess (–)	Chewiness (g)
Cow's milk	Control	89.6 ± 2.9^d^	−21.6 ± 0.8^d^	1,738.56 ± 58.07^f^	1,088.01 ± 36.29^e^
	0.1% QSSG	113 ± 3.1^b^	−34.8 ± 0.9^g^	3,005.80 ± 82.71^c^	2,167.17 ± 59.63^b^
	0.2% QSSG	124.6 ± 0.8^a^	−53.8 ± 1.5^i^	3,587.52 ± 21.99^a^	3,160.60 ± 19.37^a^
	0.1% CSG	101.9 ± 1.7^c^	−29.2 ± 0.3^e^	2,292.97 ± 38.48^d^	1,469.79 ± 24.66^d^
	0.2% CSG	115.5 ± 3.4^b^	−38.8 ± 0.8^h^	3,313.89 ± 96.31^b^	2,193.79 ± 63.76^b^
Camel milk	Control	46.5 ± 1.2^g^	−10.3 ± 0.1^a^	325.73 ± 8.40^j^	90.22 ± 2.33^i^
	0.1% QSSG	63.9 ± 0.3^f^	−18.2 ± 0.5^c^	1,041.02 ± 4.10^h^	647.51 ± 2.55^g^
	0.2% QSSG	105.2 ± 4.1^c^	−30.7 ± 1.2^f^	2,125.71 ± 83.68^e^	1,800.47 ± 70.88^c^
	0.1% CSG	62.8 ± 0.5^f^	−16.1 ± 0.2^b^	797.64 ± 6.45^i^	482.56 ± 3.90^h^
	0.2% CSG	70.7 ± 0.9^e^	−20.7 ± 0.6^d^	1,370.93 ± 17.38^g^	937.71 ± 11.89^f^

Note

In each column, means with same superscripts had no significant difference with each other (*p* > .05).

#### 3.5.2 Adhesiveness

Adhesiveness is defined as the amount of required work in order to overcome the attractive forces among the surface of the food product and the surface of the material with which it comes in contact (Bahramparvar et al., 2013). Except than the control sample in cow's frozen yoghurt and camel frozen yoghurt containing 2% CSG, gums have a significant effect on adhesiveness in other treatments (*p* < .05). Similar to the hardness, enhancement in gum concentration lead to the increase of the adhesiveness in produced frozen yoghurts. This finding is in agreement with the results of Milani and Koocheki (2011) who also concluded that the presence of guar gum in yoghurt formulation enhanced the adhesiveness in produced frozen yoghurt samples. The main reason for this phenomenon can be related to the formation of a viscous gel matrix. Also, El‐Nagar et al. (2002) stated that stickiness in frozen yoghurt was attributed to the gum concentration. Results of adhesiveness of the frozen yoghurts (produced from camel milk and cow's milk) showed that samples containing both gums were significantly more stick than control samples (Table 3). On the other hand, results showed that the adhesiveness of frozen yoghurt samples produced by QSSG was higher than produced sample from CSG (in the equal values in frozen yoghurt made with camel milk).

#### 3.5.3 Gumminess

Gumminess is defined as the required energy to disintegrate a semisolid product to make it ready for swallowing. Gumminess is unattractive parameter that affects the appearance and texture of dairy desserts such as ice cream and frozen yoghurt (Bahramparvar et al., 2013). Results showed that, the gumminess of samples, increased noticeably by the addition of gums to the frozen yoghurt formulation (Goff & Hartel, 2013). Similar results have also been reported in the case of frozen yoghurt produced by xanthan gum and hydroxylpropyl methylcellulose (Soukoulis et al., 2010).

In addition, the effect of both gums on the increase of gumminess in cow's frozen yoghurt was higher than camel frozen yoghurt (with equal values). About this subject, Varelaet al. (2014) reported that correlation among gumminess and the viscosity of the samples containing guar gum leads to an increase in samples gumminess (Table 3). According to the obtained results from the gumminess, it could be concluded that it linked to the strong textural network of the produced frozen yoghurt samples from cow's milk compared to produce samples from camel milk.

#### 3.5.4 Chewiness

Chewiness is defined as the required energy to chew a solid food product until it is ready for swallowing. As can be seen from Table 3, chewiness increased with increasing both gum concentrations in frozen yoghurts. The chewiness was obtained in the range of 90.22–3,160.60 g, related to the camel frozen yoghurt without gum and cow's frozen yoghurt containing 0.2% QSSG, respectively (Table 3). In this study, a significant increase (*p* < .05) was observed in chewiness for all samples except the samples made from cow's milk containing 0.1% QSSG and 0.2% CSG that were not significant. Wittinger and Smith (1986) also reported that the presence of locust bean and guar gums interacted to enhance chewiness of the ice cream. Based on these results, excessive use of stabilizer leads to increasing gumminess, in which the product cannot melt quickly in the mouth and retains excessive chewiness (Goff & Hartel, 2013).

### 3.6 Sensory evaluation

The results obtained from the data mean comparison of sensory attributes are illustrated in Table 4. Results showed that the addition of QSSG and CSG had a significant effect on the color, flavor, texture and total acceptance of the frozen yoghurt samples. Moeenfard and Tehrani (2008) and Rezaei et al. (2011) studied the relationship between the type and concentration of stabilizers with viscosity, body and texture, and total acceptance of the produced ice cream. These researchers stated that the type and concentration of stabilizers were highly affected the viscosity, body and texture of frozen yoghurt mixtures. The results of other studies also showed that QSSG and CSG improved the rheological attributes of foods and provide the suitable texture and mouth feel for industrial application (Behrouzian et al., 2014; Behrouzian, Razavi, & Phillips, 2014; Koocheki et al., 2013). However, the increase of the QSSG and CSG gum concentration lead to increase in the sensory scores of color, texture and total acceptability but in lower concentration these differences were not significant.

**TABLE 4:** Sensory analysis results for the cow's and camel frozen yoghurt samples with QSSG and CSG

Frozen yoghurt	Gum concentration (%)	Color	Texture	Flavor	Total
Cow's milk	Control	5 ± 0.00^a^	3 ± 0.00^cde^	5 ± 0.00^a^	3 ± 0.00^bc^
	1% QSSG	4.33 ± 0.58^ab^	4 ± 0.00^b^	4.33 ± 0.58^ab^	4 ± 0.00^a^
	2% QSSG	3.33 ± 0.58^b^	5 ± 0.00^a^	3 ± 0.00cd^cde^	3.67 ± 0.58^ab^
	1% CSG	4 ± 0.00^b^	3.67 ± 0.58^bc^	4.66 ± 0.58^a^	3.67 ± 0.58^ab^
	2% CSG	3.67 ± 0.58^b^	4 ± 0.00^b^	3.67 ± 0.57^bc^	4 ± 0.00^a^
Camel milk	Control	5 ± 0.00^a^	2.33 ± 0.58^e^	3.33 ± 0.58^cd^	2.67 ± 0.58^d^
	1% QSSG	4 ± 0.00^b^	3.33 ± 0.58^bcd^	2.67 ± 0.58^de^	2.67 ± 0.58^c^
	2% QSSG	3.67 ± 0.00^b^	4 ± 0.00^b^	2.67 ± 0.58^de^	3 ± 0.00b^c^
	1% CSG	4.33 ± 0.58^ab^	2.67 ± 0.58^de^	3.33 ± 0.58^cd^	2.67 ± 0.58^c^
	2% CSG	4 ± 0.00^b^	3.33 ± 0.58^bcd^	2.33 ± 0.58^e^	3 ± 0.00b^c^

Note

In each column, means with same superscripts had no significant difference with each other (*p* > .05).

As can be seen from Table 4, camel frozen yoghurt has different sensory properties, particularly in term of flavor and texture compared to cow's frozen yoghurt. Al‐Saleh et al. (2011) by investigating sensory properties of frozen yoghurt made from camel milk showed that unpleasant flavor of camel frozen yoghurt was observed easier than cow's frozen yoghurt. In fact, the results obtained will validate this subject. In general, cow's frozen yoghurts containing 2% CSG and 1% QSSG (score = 4) were more acceptable among panelists than camel frozen yoghurt.

## 4 CONCLUSIONS

The results showed that the addition of QSSG and CSG to yoghurts mixture improved the viscosity, first dripping times, complete melting times, textural, and sensory properties, but had no significant effect on the pH and acidity of the final product. Therefore, the gums obtained from *Lepidium perfoliatum* and *Lepidium sativum* seeds have the potential to be applied as a new source of polysaccharides for producing frozen yoghurts with improved properties. The cow's frozen yoghurt mixture had the higher apparent viscosity than the camel frozen yoghurt mixture at the equal concentration of gums. This is an indication that frozen yoghurt mixes are non‐Newtonian at all added concentrations. Along with increase in gum concentration, the rheological characteristics of frozen yoghurt mixes were changed from Newtonian flow behavior to non‐Newtonian flow behavior. Results showed that the addition of QSSG and CSG had a significant effect on the color, flavor, texture, and total acceptance of the frozen yoghurt samples and by the increase of the QSSG and CSG gum concentration, sensory scores of cow's frozen yoghurt samples were higher than camel frozen yoghurt samples.

## CONFLICT OF INTEREST

The authors declare that they do not have any conflict of interest.

## ETHICAL APPROVAL

This study does not involve any human or animal testing.

## INFORMED CONSENT

Written informed consent was obtained from all study participants.

## Data Availability

Research data are not shared.
